# Overexpression of the transcription factor NF-YC9 confers abscisic acid hypersensitivity in *Arabidopsis*

**DOI:** 10.1007/s11103-017-0661-1

**Published:** 2017-09-18

**Authors:** Chao Bi, Yu Ma, Xiao-Fang Wang, Da-Peng Zhang

**Affiliations:** MOE Systems Biology and Bioinformatics Laboratory, Center for Plant Biology, School of Life Sciences, Tsinghua University, Beijing, 100084 China

**Keywords:** Abscisic acid signaling, *Arabidopsis thaliana*, Nuclear factor NF-YC9, ABA-INSENSITIVE5 (ABI5), Seedling growth, Stomata

## Abstract

Nuclear factor Y (NF-Y) family proteins are involved in many developmental processes and responses to environmental cues in plants, but whether and how they regulate phytohormone abscisic acid (ABA) signaling need further studies. In the present study, we showed that over-expression of the *NF-YC9* gene confers ABA hypersensitivity in both the early seedling growth and stomatal response, while down-regulation of *NF-YC9* does not affect ABA response in these processes. We also showed that over-expression of the *NF-YC9* gene confers salt and osmotic hypersensitivity in early seedling growth, which is likely to be directly associated with the ABA hypersensitivity. Further, we observed that NF-YC9 physically interacts with the ABA-responsive bZIP transcription factor ABA-INSENSITIVE5 (ABI5), and facilitates the function of ABI5 to bind and activate the promoter of a target gene *EM6*. Additionally, NF-YC9 up-regulates expression of the *ABI5* gene in response to ABA. These findings show that NF-YC9 may be involved in ABA signaling as a positive regulator and likely functions redundantly together with other NF-YC members, and support the model that the NF-YC9 mediates ABA signaling via targeting to and aiding the ABA-responsive transcription factors such as ABI5.

**Electronic supplementary material:**

The online version of this article (doi:10.1007/s11103-017-0661-1) contains supplementary material, which is available to authorized users.

## Introduction

The phytohormone abscisic acid (ABA) regulates plant growth, development, and responses to a variety of abiotic stress (Finkelstein et al. 2002; Adie et al. 2007; Cutler et al. 2010). Numerous regulators of ABA signaling, including receptors for ABA, have been identified, revealing the functional mechanism of this phytohormone from signal perception to downstream gene expression (for review, see Cutler et al. 2010). However, given that ABA signaling is a highly complex signaling pathway, many other components remain to be identified to fully understand the complex ABA signaling network.

Nuclear factor Y (NF-Y), also called CCAAT box combination factor (CBF) or heme activated protein (HAP), is a class of transcription factors that widely exist in yeast, animals and plants. NF-Y consists of three different subunits, the NF-YA (CBF-B or HAP2), NF-YB (CBF-A or HAP3) and NF-YC (CBF-C or HAP5) (Mantovani 1999). In plants, each of the three subunits has multiple members (Stephenson et al. 2007; Siefers et al. 2009; Laloum et al. 2013). In *Arabidopsis thaliana*, there are ten members of NF-YA subfamily, 13 members of NF-YB subfamily, and 13 members of NF-YC subfamily (Siefers et al. 2009). The NF-YA, NF-YB and NF-YC subunits usually form a huge number of hetero-trimer complex, which functions to bind DNA and regulate gene expression (Gusmaroli et al. 2001; Petroni et al. 2012). It has been believed that, while the NF-Y proteins have retained high degrees of similarity, especially in the residues necessary for NF-Y complex formation and DNA binding, they may be evolving unique, even antagonistic, regulatory roles for some processes likely through formation of different complexes with different NF-Y members (Stephenson et al. 2007; Siefers et al. 2009; Laloum et al. 2013). While the NF-Y family proteins function as a hetero-trimer complex, a recent report showed that *Arabidopsis* NF-YC1/HAP5A directly bound the promoter of a target gene regulated the gene expression in freezing response (Shi et al. 2014), revealing that a single NF-Y subunit may function alone without formation of a hetero-trimer complex. Additionally, increasing evidence showed that the NF-Y members may work together or alone to interact with and help other transcription factors to function in gene expression regulation (Wright et al. 1995; Benatti et al. 2008; Yamamoto et al. 2009; Liu and Howell 2010; Kumimoto et al. 2013; Yotsui et al. 2013; Yeap et al. 2017). Recent reports showed that NF-Y members are involved in chromatin remodeling (Cao et al. 2014; Hou et al. 2014; Tang et al. 2017). These findings further support the functional diversity and complexity of the NF-Y family proteins.

Recent studies showed importance of the NF-Y family proteins in ABA signaling (Warpeha et al. 2007; Yamamoto et al. 2009; Kumimoto et al. 2013; Shi et al. 2014; Siriwardana et al. 2014; Liu et al. 2016) and ABA-mediated stress responses (Nelson et al. 2007; Li et al. 2008, 2013; Leyva-González et al. 2012; Sato et al. 2014; Shi et al. 2014). NF-YA5 mutants were shown to be hypersensitive to ABA during seed germination (Warpeha et al. 2007) and susceptible to drought, but overexpression of NF-YA5 increased drought tolerance in an ABA-dependent manner (Li et al. 2008). In a complete NF-Y family analysis, all the ten *Arabidopsis* NF-YA members were systematically over-expressed, and the results showed that some transgenic lines were hypersensitive and others were hyposensitive to ABA in seed germination, in which the overexpression lines of NF-YA2, NF-YA4, NF-YA5, NFYA7, NF-YA8, and NF-YA10 were ABA hypersensitive, and those of NF-Y1 and NFYA9 were ABA hyposensitive (Leyva-González et al. 2012; Mu et al. 2013). The opposing ABA-related phenotypes suggest that this closely-related homologues evolved distinct roles during ABA-mediated seed germination (Siriwardana et al. 2014). The NF-YC members were also recently reported to be involved in ABA signaling. Different NF-YC proteins were shown to have unique and opposing functions in ABA-mediated seed germination, in which mutants of NF-YC4 were hypersensitive, but mutants of NF-YC3 were hyposensitive, to ABA, and mutants of NF-YC9 displayed wild-type ABA-related phenotype, during seed germination (Kumimoto et al. 2013). However, a most recent report showed that the seeds of an NF-YC9- overexpression line have lower testa and endosperm rupture rate, showing that overexpression of NF-YC9 results in ABA hypersensitivity in seed germination (Liu et al. 2016). Therefore, whether and how NF-Y family proteins regulate ABA signaling needs further studies to understand the complex mechanism of ABA function.

In the present study, we showed that overexpression of NF-YC9 enhances the sensitivity to ABA and salt and osmotic stresses during early seedling growth, and also promotes stomatal response to ABA, though the knockdown mutants of NF-YC9 show wild-type ABA-related phenotypes, suggesting that NF-YC9 may positively regulate ABA signaling but likely with a functional redundancy. We further showed that NF-YC9 interacts with and improves activities of an ABA responsive bZIP transcription factor ABI5 and enhances expression of *ABI5* gene in response to ABA. These findings help to understanding functional role of the NF-YC proteins in ABA signaling.

## Materials and methods

### Plant materials and growth conditions

*Arabidopsis thaliana* ecotype Col-0 was used as the wild type materials. The mutants *nf-yc9-1* (SALK_058903) and *nf-yc9-2* (SALK_069632) were obtained from the Arabidopsis Biological Resource Center (ABRC, Columbus, OH, USA). The homozygous mutants were identified by PCR using gene-specific primers (see Supplemental Table S1 in Supplemental Data). The mutant *nf-yc9-1* and the *NF-YC9* overexpression transgenic lines OE1 and OE6, generous gifts from Dr. Hao Yu (Department of Biological Sciences, National University of Singapore), were previously identified and described (Hou et al. 2014). We verified the *NF-YC9* mRNA levels in the *nf-yc9* mutants and *NF-YC9* overexpression lines OE1 and OE6 by quantitative RT-PCR analysis with the gene-specific primers ( see Supplemental Table S2 in Supplemental Data).

Plants were grown in a growth chamber at 22 °C on MS medium (Phyto Technology Laboratories, Shawnee Mission, KS, USA, product No. M519) in a growth chamber at ~80 μmol photons m^−2^ s^−1^ or in compost soil at ~120 μmol photons m^−2^ s^−1^ lighted by cool-white fluorescent lamps under a 16 h-light/8 h-dark photoperiod and 60% relative humidity.

### Phenotypic analysis

Phenotypic analysis was performed as described previously (Wu et al. 2009; Shang et al. 2010). For the post-germination growth and cotyledon greening assays, seeds (about 100) were sterilized and plated on MS medium that contained 3% sucrose and 0.8% agar (pH 5.9) supplemented with different concentrations of ABA or NaCl or d-mannitol (Amresco, Solon, OH, USA). The seeds were chilled at 4 °C for 3 days before being placed at 22 °C under light conditions (16-h light/8-h dark). The fresh weight of single seedling, the root length and the cotyledon greening rate were measured at the indicated times. Leaves of the 4-week-old seedlings grown in the soil were used for investigation of the stomatal closure. To observe ABA-induced stomatal closure, detached leaves were immersed in a buffer containing 50 mM KCl and 10 mM MES-KOH (pH 6.15) under a halogen cold light source (Chongqing Optec Instrument Co., Lt, Chongqing, China) for 3 h. Subsequently, the detached leaves were transferred into the fresh buffer solution (50 mM KCl and 10 mM MES-KOH pH 6.15) with 20 μM (±) ABA (Sigma, Saint Louis, MO, USA) for 2 h before the stomatal apertures were measured. To assay ABA-inhibited stomatal opening, the plants were kept in the dark for 24 h before the detached leaves were immersed in the same buffer described as above supplemented with 20 μM (±) ABA for 2 h under the cold light, and the apertures were measured. In addition, epidermal strips were removed from the abaxial surface of the detached leaves and photographed with the Olympus DIC microscope (Olympus, BX51, Japan).

### Yeast two-hybrid assay

The interaction between NF-YC9 and ABI5 proteins was assayed by a yeast two-hybrid system, of which the experimental procedures were described by the manufacturer (Clontech, Mountain View, CA, USA). The full-length coding sequence of *NF-YC9* was cloned to pGADT7 vector fused with the DNA activation domain (AD), and the full-length coding sequence of *ABI5* was cloned to pGBKT7 vector fused to GAL4 binding domain (BD). The primers used for the related constructs are listed in Supplemental Table S3. The different combination pairs of the constructs were used to co-transform yeast strain AH109. Co-transformants were plated on Leu-Trp-deficient medium (SD-2) and Leu-Trp-His-Ade-deficient medium (SD-4) and grown for 5–7 days at 30 °C. The yeast cells co-transformed with the construct pairs AD plus BD-ABI5 and BD plus AD-NF-YC9 were used as negative controls, and the yeast cells co-transformed with the construct pairs BD-p53 plus AD-T were taken as a positive control.

### Electrophoretic mobility shift assay (EMSA)

EMSA was performed essentially as previously described (Liu et al. 2012, 2013; Shang et al. 2010) and by using the Light Shift Chemiluminescent EMSA kit (Thermo Scientific, Waltham, MA, USA, product No. 89,880) according to the manufacturer instructions. The NF-YC9-6His and ABI5-6His recombinant proteins were produced in *E. coli* strain BL21. The full-length coding sequence of the *NF-YC9* or *ABI5* gene was cloned into the Sal I/EcoR I sites of pMAL-c5X vector (NEB), respectively. The 5′ end biotin-labeled probes used for this EMSA were amplified by PCR using the primer pairs described in Supplemental Table S4, and unlabeled fragments of the same sequences were used as the competitors. The labeled probes (20 fmol) were incubated with the ABI5-6His recombinant proteins (1 µg) in a binding buffer solution (25 mM HEPES, 40 mM KCl, 5 mM MgCl_2_, 1 mM DTT, 1 mM EDTA, and 8% glycerol, pH 8.0) in the presence of 1 µg µL^−1^ of poly(deoxyinosinic-deoxycytidylic) sodium salt [poly(dI-dC)] (Sigma, USA, product No. P4929) for 30 min at room temperature. The NF-YC9-6His protein was applied at a gradient of concentrations (1–4 μg). The DNA–protein reaction mixtures were separated on a 5% non-denaturing polyacrylamide gel (acrylamide:bisacrylamide = 19:1). The detailed procedures were described in the manufacturer instructions of the EMSA kit.

### Luciferase complementation imaging (LCI)

LCI assay was performed to further verify the interaction between NF-YC9 and ABI5 in *Nicotiana benthamiana* leaves according to previously described procedures (Chen et al. 2008; Shang et al. 2010). The full-length coding sequence of *NF-YC9* and *ABI5* were constructed to the C-terminus of Luc (CLuc) vector and the N-terminus of Luc (NLuc) vector, respectively. Primers used for the vector construction were listed in Supplemental Table S5. The constructs were transformed into *Agrobacterium tumefaciens* GV3101. Bacteria were suspended in the infiltration buffer solution (0.2 mM acetosyringone, 10 mM MgCl2, and 10 mM MES) to identical concentrations (OD600 = 0.6). Bacterial suspensions were infiltrated into young but fully expanded leaves of the seven-week old *N. benthamiana* leaves using a needleless syringe. It is noteworthy that the amounts of the constructs were the same between the different treatments and controls for each group of assay. After infiltration, plants were grown firstly under dark for 24 h and then with 16 h light/day for 60 h at room temperature. The luciferase (LUC) activity was observed with a CCD imaging apparatus (Andor iXon, Andor Technology, Belfast, UK). The leaves were sprayed with 0.5 mM luciferin and were placed in darkness for 3 min before luminescence detection.

### Assay of the transcription factor-promoter interaction in *N. benthamiana* leaves

This assay was performed essentially as previously described (Shang et al. 2010). The NF-YC9 and ABI5 proteins were used for the effectors. The promoter of the *EM6* gene was amplified by PCR using the primer pairs listed in Supplemental Table S6. The PCR product was cloned to generate the reporter construct, promoter of *EM6* linked to luciferase (ProEM6::LUC). The full-length coding sequence of the *NF-YC9* and *ABI5* gene were fused to the pMDC85 and pCAMBIA1300-221 vector as the effector constructs (35S::NF-YC9, 35S::ABI5), which were used to transform, respectively, *A. tumefaciens strain* GV3101. The transformant-bacteria suspensions were infiltrated into the 7-week old *N. benthamiana* plants. It is noteworthy that the amounts of the *A. tumefaciens* strain containing different constructs were the same between the different treatments and controls for each group of assay. After infiltration, plants were grown firstly under dark for 24 h and then with 16 h light/day for 60 h at room temperature. The LUC activity was observed with a CCD imaging apparatus (Andor iXon, Andor Technology, Belfast, UK). The leaves were sprayed with 0.5 mM luciferin and were placed in darkness for 3 min before luminescence detection.

### Bimolecular imaging of fluorescent complementation (BiFC) assay

BiFC assays were performed essentially as previously described (Shang et al. 2010). We used the wild type Col-0 plants to prepare Arabidopsis protoplast. The full-length coding sequences of *NF-YC9* and *ABI5* were constructed to the pUC-SPYNE and pUC-SPYCE vector (cYFP-NF-YC9 or ABI5-nYFP), respectively. The primers used for constructing the related plasmids are listed in Supplemental Table S7. The YFP fluorescence was imaged under a Leica confocal laser scanning microscope (Zeiss LSM780, Germany).

### Transient expression in *Arabidopsis* protoplasts to assay subcellular localization of NF-YC9 protein

Transient expression in the *Arabidopsis* protoplasts was performed essentially as previously described (Shang et al. 2010). NF-YC9 was fused with the green fluorescent protein (GFP) protein (NF-YC9-GFP), and the known nuclear- and cytoplasmic protein PYR1 (Park et al. 2009) was fused with the red fluorescent protein (RFP) as a control (PYR1-RFP). The corresponding cDNAs were amplified by PCR (see Supplemental Table S8 for the primers). Protoplasts were isolated from the leaves of 3- to 4-week old plants of *Arabidopsis* (ecotype Col-0). Fluorescence of GFP or RFP was observed by confocal microscope (Zeiss LSM780, Germany).

### Quantitative RT-PCR

For quantitative RT-PCR (qRT-PCR) analysis, total RNA was isolated from the 3-weeks-old seedlings. Seeds were sown in MS medium, chilled for 3 days at 4 °C and placed at 22 °C under a 16-h light/8-h dark photoperiod for 3 weeks. Using Total RNA Rapid Extraction Kit (BioTeke, Beijing, China, product No. RP3301), the RNA samples for qRT-PCR were prepared, treated with RNase-free DNaseI (NEB) at 37 °C for 30–60 min to degrade genomic DNA, and purified by using RNA Purification Kit (BioTeke, Beijing, China, product No. RP1801). Total RNA (2 µg) were subjected to first-strand cDNA synthesis using a kit according to the manufacturer’s instructions (Roche, Mannheim, Germany, product No.AS095014379012001). The primers used for qRT-PCR are listed in Supplemental Table S9. Analysis was performed using the Bio-Rad Real-Time System CFX96TM C1000 Thermal Cycler (Bio-Rad, USA). Amplification of the *Actin 2*/*8* genes was used as an internal control. The qRT-PCR conditions were as follows: 10 min of denaturation at 95 °C, followed by 45 cycles of 10 s of denaturation at 95 °C, 5 s of annealing at 55 °C and a 10 s extension at 72 °C. The cDNA was amplified using SYBR Premix ExTaq (TaKaRa, Dalian, China). All experiments were repeated at least three times along with three biologically independent repetitions.

## Results

### Over-expression of *NF-YC9* results in ABA hypersensitivity in early seedling growth and stomatal response

**Fig. 1:**
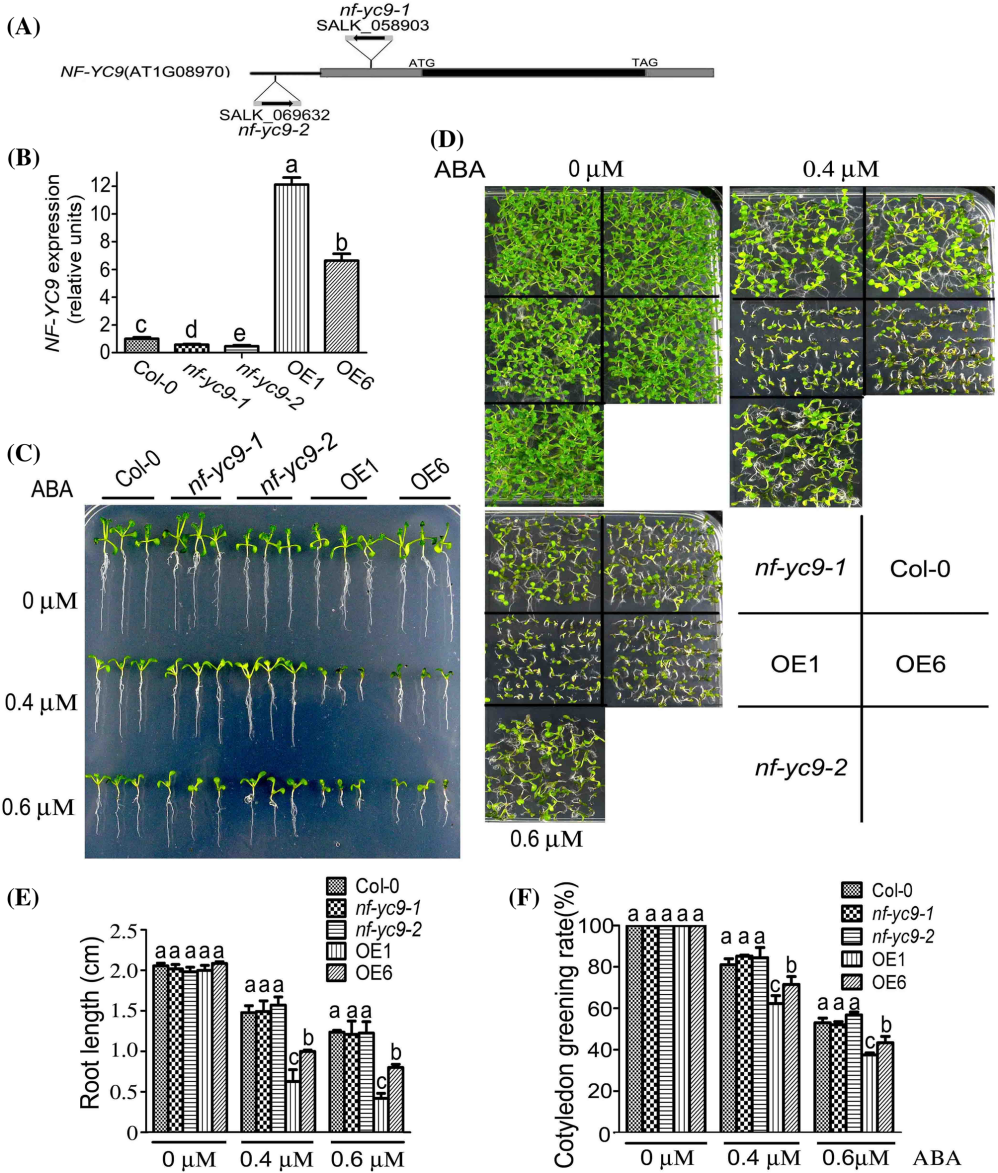
Over-expression of *NF-YC9* results in ABA hypersensitivity but down-regulation of *NF-YC9* expression shows no effect on ABA response during early seedling growth. **a** Structures of the *NF-YC9* gene are shown with the T-DNA insertion site in *nf-yc9-1* (SALK_058903) and *nf-yc9-2* (SALK_069632). Black box: exon; grey box: 5′ and 3′ UTR; thick line: the promoter region from the start code ATG of the *NF-YC9* gene. The black arrowhead indicates the orientation of the T-DNA insertion. **b** Quantitative RT-PCR analysis of *NF-YC9* gene expression in wild-type Col-0, *nf-yc9-1, nf-yc9-2* mutants and the *NF-YC9* overexpression transgenic lines OE1 and OE6. Expression level of *NF-YC9* in all the genetic materials was normalized to that of *Actin2*/*8*, and the expression level of *NF-YC9* in the wild-type Col-0 was set to 1. Each value is the mean ± SE of three independent biological experiments, and different letters indicate significant differences at P < 0.05 (Duncan’s multiple range test). **c** Root growth of the different genotypes (the wild-type Col-0, *nf-yc9-1*and *nf-yc9-2* knockdown mutants, and the *NF-YC9* overexpression transgenic lines OE1 and OE6) growing on (±)ABA-free (0 μM) and ABA-containing (0.4 and 0.6 μM) MS medium. Seeds were directly sown on the medium, and the root growth was investigated 14 days after a stratification at 4 °C for 3 days. The experiments were repeated four times with similar results. **d** Post-germination growth of the different genotypes as shown in (**c**). Seeds were directly sown on (±)ABA-free (0 μM) or ABA-containing (0.4 and 0.6 μM) MS medium, and the growth was investigated 10 days after a stratification at 4 °C for 3 days. The experiments were repeated four times with similar results. **e** Statistical analysis of root length of the different genotypes described in (**c**). Each value is the mean ± SE of three independent biological experiments, and different letters indicate significant differences at P < 0.05 (Duncan’s multiple range test) when comparing values within the same ABA concentration. **f** Statistical analysis of cotyledon greening rate of the different genotypes as described in (**d**). Green cotyledons were recorded 7 days after a stratification at 4 °C for 3 days. Each value is the mean ± SE of three independent biological experiments, and different letters indicate significant differences at P < 0.05 (Duncan’s multiple range test) when comparing values within the same ABA concentration

The T-DNA insertion homozygous mutants of the *NF-YC9* gene, *nf-yc9-1* and *nf-yc9-2*, were shown to be knockdown alleles (Fig. 1a, b). The *NF-YC9* gene was significantly over-expressed in the transgenic lines OE1 and OE6 (Fig. 1b). We observed that, in the absence of exogenously applied ABA, there was no significant difference found between the wild-type and *NF-YC9*-overexpressing lines (OE1 and OE6) plants in the early seedling growth (as indicated by both primary root length and cotyledon greening rate), while the early seedling growth of the OE1 and OE6 lines was significantly more reduced than that of the wild-type seedlings in the ABA-containing medium (Fig. 1c–f).

**Fig. 2:**
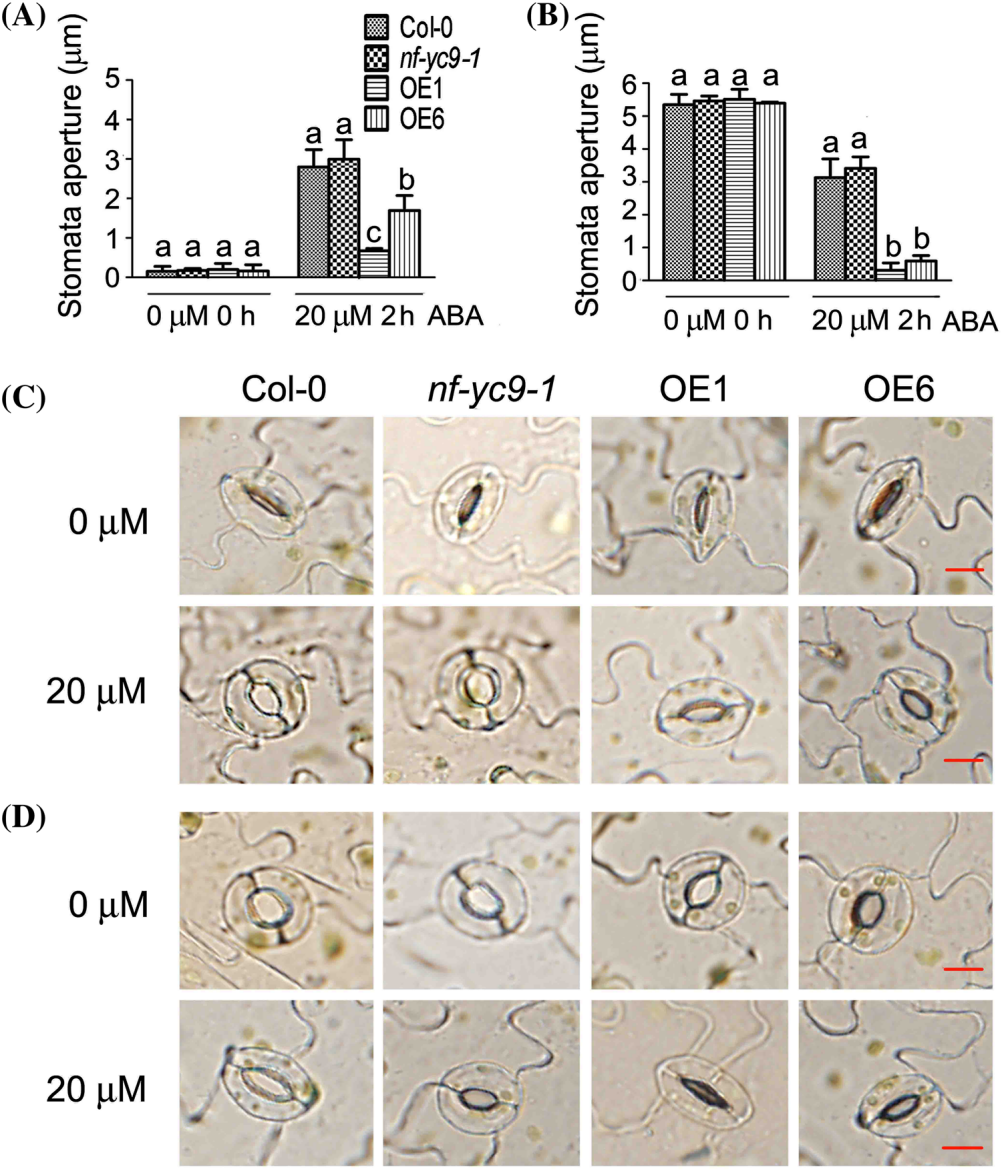
Over-expression of *NF-YC9* promotes, but down-regulation of *NF-YC9* expression shows no effect on, stomatal response to ABA. **a** ABA inhibition of stomatal opening, and **b** ABA promotion of stomatal closure, in the wild-type Col-0, *nf-yc9-1* knockdown mutant and the *NF-YC9* overexpression transgenic lines OE1 and OE6. Mature rosette leaves from 3-week-old seedlings were used for the assays. Each value is the mean ± SE of three independent biological experiments, and different letters indicate significant differences at P < 0.05 (Duncan’s multiple range test) when comparing values within the same ABA concentration. **c** Photographs showing the stomatal aperture of the ABA-induced inhibition of stomatal opening in the different genotypes as described in (**a**). Bars represent 10 μm. **d** Photographs showing the stomatal aperture of the ABA-induced promotion of stomatal closure in the different genotypes as described in (**b**). Bars represent 10 μm

The promotion of stomatal closure and inhibition of stomatal opening have been believed to be two distinct ABA-mediated processes, which can minimize water transpiration from the leaves under drought conditions (Zhu 2002; Kwak et al. 2008). We observed that, in the presence of exogenously applied ABA, the OE1 and OE6 plants exhibited significantly ABA-hypersensitive phenotypes in both ABA-induced promotion of stomatal closure and inhibition of stomatal opening compared with wild-type plants (Fig. 2a–d).

These data suggest that NF-Y9 may be positively involved in ABA signaling in both early seedling growth and stomatal response. However, we found that the knockdown mutants *nf-yc9-1*and *nf-yc9-2* displayed wild-type phenotypes in early seedling growth and stomatal response (Figs. 1, 2), suggesting that the *Arabidopsis* NF-YC family proteins may function redundantly in these ABA-mediated processes.

### Over-expression of *NF-YC9* leads to salt and osmotic hypersensitivity during early seedling growth

**Fig. 3:**
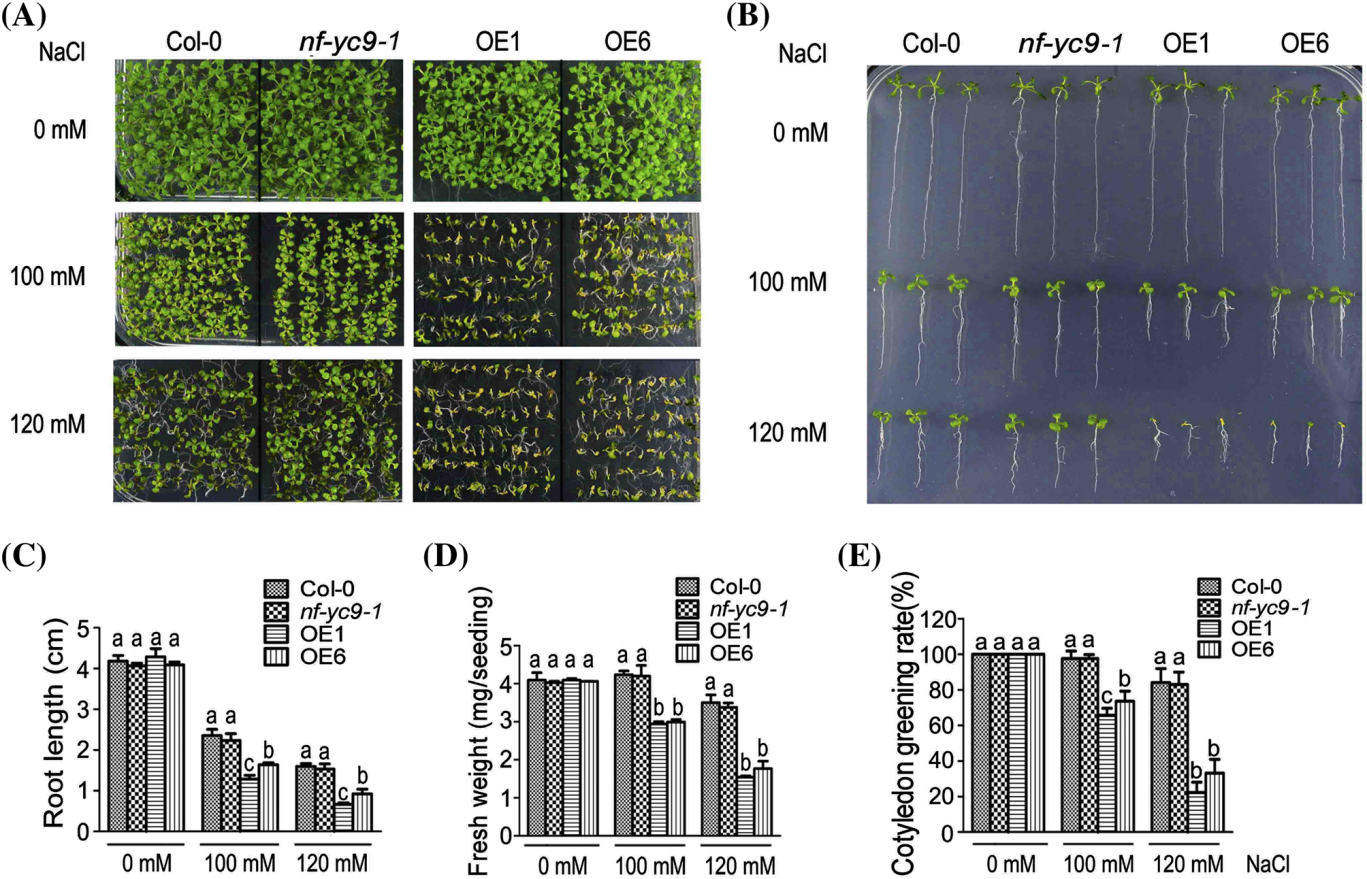
Over-expression of *NF-YC9* results in salt stress hypersensitivity but down-regulation of *NF-YC9* expression shows no effect on salt response during post-germination growth. **a** Post-germination growth of the wild-type Col-0, *nf-yc9-1* knockdown mutant and the *NF-YC9* overexpression transgenic lines OE1 and OE6 plants. Seeds were directly sown on NaCl-free (0 mM) or NaCl-containing (100 mM, and 120 mM) MS medium, and the growth was investigated 10 days after a stratification at 4 °C for 3 days. The experiments were repeated four times with similar results. **b** Root growth of the different genotypes as described in (**a**) growing on NaCl-free (0 mM) or NaCl-containing (100, and 120 mM) MS medium. Seeds were directly sown on the medium, and the root growth was investigated 14 days after a stratification at 4 °C for 3 days. The experiments were repeated four times with similar results. **c** Statistical analysis of root length of the different genotypes as described in (**b**). Each value is the mean ± SE of three independent biological experiments, and different letters indicate significant differences at P < 0.05 (Duncan’s multiple range test) when comparing values within the same NaCl concentration. **d** Statistical analysis of fresh weight (mg/seedling) of the different genotypes as described in (**a**). Each value is the mean ± SE of three independent biological experiments, and different letters indicate significant differences at P < 0.05 (Duncan’s multiple range test) when comparing values within the same NaCl concentration. **e** Statistical analysis of cotyledon greening rate of the different genotypes as described in (**a**). Green cotyledons were recorded 7 days after a stratification at 4 °C for 3 days. Each value is the mean ± SE of three independent biological experiments, and different letters indicate significant differences at P < 0.05 (Duncan’s multiple range test) when comparing values within the same NaCl concentration

**Fig. 4:**
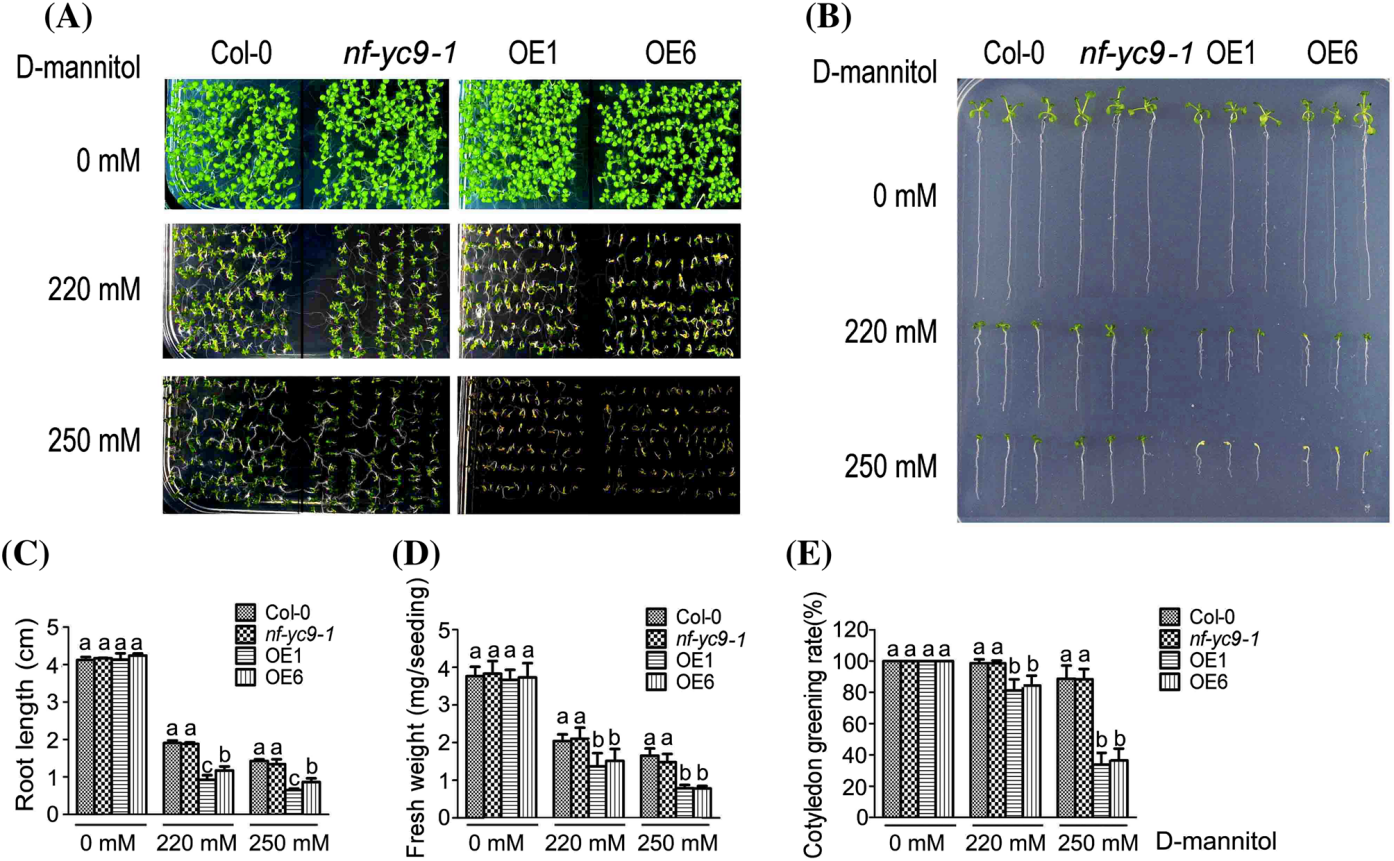
Over-expression of *NF-YC9* results in osmotic stress hypersensitivity but down-regulation of *NF-YC9* expression shows no effect on osmotic response during post-germination growth. **a** Post-germination growth of the wild-type Col-0, *nf-yc9-1* knockdown mutant and the *NF-YC9* overexpression transgenic lines OE1 and OE6 plants. Seeds were directly sown on d-mannitol-free (0 mM) or d-mannitol-containing (220, and 250 mM) MS medium, and the growth was investigated 10 days after a stratification at 4 °C for 3 days. The experiments were repeated four times with similar results. **b** Root growth of the different genotypes as described in (**a**) growing on d-mannitol-free (0 mM) or d-mannitol-containing (220, and 250 mM) MS medium. Seeds were directly sown on the medium, and the root growth was investigated 14 days after a stratification at 4 °C for 3 days. The experiments were repeated four times with similar results. **c** Statistical analysis of root length of the different genotypes as described in (**b**). Each value is the mean ± SE of three independent biological experiments, and different letters indicate significant differences at P < 0.05 (Duncan’s multiple range test) when comparing values within the same d-mannitol concentration. **d** Statistical analysis of fresh weight (mg/seedling) of the different genotypes as described in (**a**). Each value is the mean ± SE of three independent biological experiments, and different letters indicate significant differences at P < 0.05 (Duncan’s multiple range test) when comparing values within the same d-mannitol concentration. **e** Statistical analysis of cotyledon greening rate of the different genotypes as described in (**a**). Green cotyledons were recorded 7 days after a stratification at 4 °C for 3 days. Each value is the mean ± SE of three independent biological experiments, and different letters indicate significant differences at P < 0.05 (Duncan’s multiple range test) when comparing values within the same d-mannitol concentration

We observed that, in the absence of exogenously applied NaCl or d-mannitol (used as a osmotic reagent), there was no significant difference found between the wild-type and *NF-YC9*-overexpressing lines (OE1 and OE6) plants in the early seedling growth (as indicated by primary root growth, plant fresh weight and cotyledon greening), whereas the early seedling growth of the OE1 and OE6 lines was significantly more reduced than that of the wild-type seedlings in the salt- or d-mannitol-containing medium (Figs. 3a–e, 4a–e). Given that both *nf-yc9* mutants showed the same phenotypes, the *nf-yc9-1* mutant was used as a representative in the following experiments, which displayed wild-type phenotypes in response to these stresses (Figs. 3a–e, 4a–e). These data are consistent with those described above for ABA responses (Figs. 1, 2), suggesting that NF-YC9 may be positively involved in both ABA- and salt-induced osmotic response during early seedling growth, and also supporting the idea that the NF-YC family proteins may function redundantly in the ABA- and salt-induced osmotic responses.

### Subcellular localization of NF-YC9 protein and expression profile of *NF-YC9* gene

**Fig. 5:**
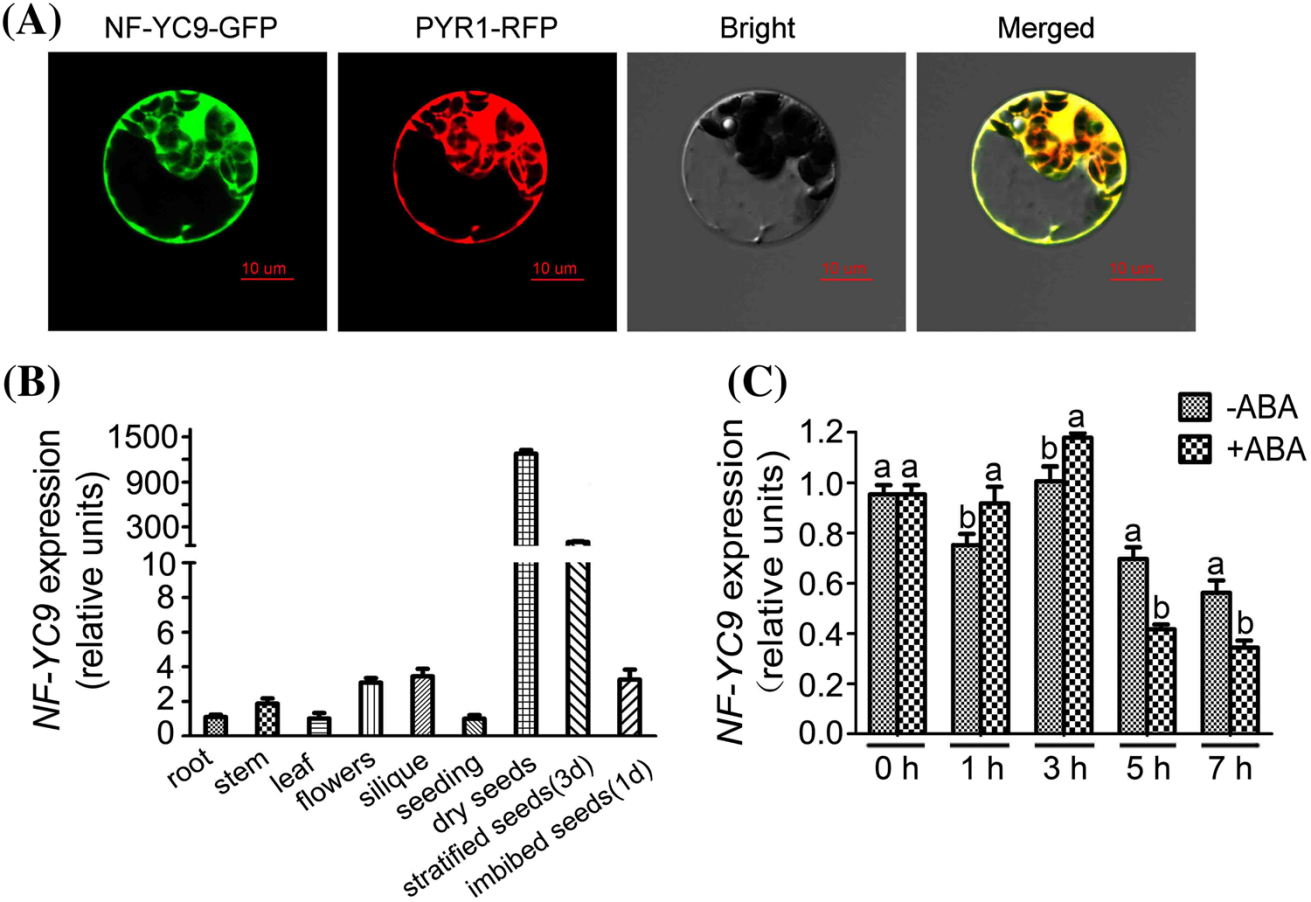
Subcellular localization of NF-YC9 protein and expression profile of *NF-YC9* gene. **a** NF-YC9 is a nuclear and cytoplasmic dual-localization protein. NF-YC9 was fused with the green fluorescent protein (GFP) protein (NF-YC9-GFP); PYR1 was fused with the red fluorescent protein (RFP) as a control (PYR1-RFP). Subcellular localization was assayed by using the transient expression system in *Arabidopsis* protoplasts. Bright, bright field; Merged: merged image of the NF-YC9-GFP and PYR1-RFP in the bright field. Bars represent 10 μm. **b** Expression profile of *NF-YC9* in various tissues/organs of wild type plants examined by real-time PCR. Relative units for the *NF-YC9* expression are normalized relative to the value of root, which is taken as 1. Each value is the mean ± SE of three independent biological experiments. **c** The expression levels of *NF-YC9* change in response to ABA treatment. Seedlings of the wild-type Col-0 plants were treated with 100 μM (±) ABA solution or mock solution [0 μM (±) ABA solution, as a control], and sampled for gene expression analysis from 0 to 7 h with the intervals of 2 h after the ABA treatment. Relative units for the gene expression are normalized relative to the value of wild-type plants at 0 h with 0 μM (±) ABA treatment, which is taken as 1. The transcript levels of *NF-YC9* were examined using quantitative RT-PCR analysis. Each value is the mean ± SE of three independent biological experiments, and different letters indicate significant differences at P < 0.05 (Student’s *t* test) when comparing values within the same time point

The transient expression assay in *Arabidopsis* protoplasts showed that NF-YC9 fused with GFP localizes to both nucleus and cytoplasm, merged well with a known nuclear- and cytoplasmic protein PYR1, which was fused with RFP and is a member of ABA receptors (Park et al. 2009) (Fig. 5a).

RT-PCR assays showed that the NF-YC9 was ubiquitously expressed in all the organs/tissues with the highest level in dry seeds (Fig. 5b), which is essentially consistent with the expression data found at the public bioinformatics websites AtGenExpress (http://jsp.weigelworld.org/expviz/expviz.jsp) and Arabidopsis eFP Browe (http://bar.utoronto.ca/efp/cgi-bin/efpWeb.cgi?dataSource=Developmental_Map) (see Supplemental Fig. S1 in Supplemental Data). Further, we observed that *NF-YC9* expression level was induced first and then repressed by ABA treatment (Fig. 5c), which may be associated with its role in ABA signaling.

### NF-YC9 interacts with ABI5 physically

**Fig. 6:**
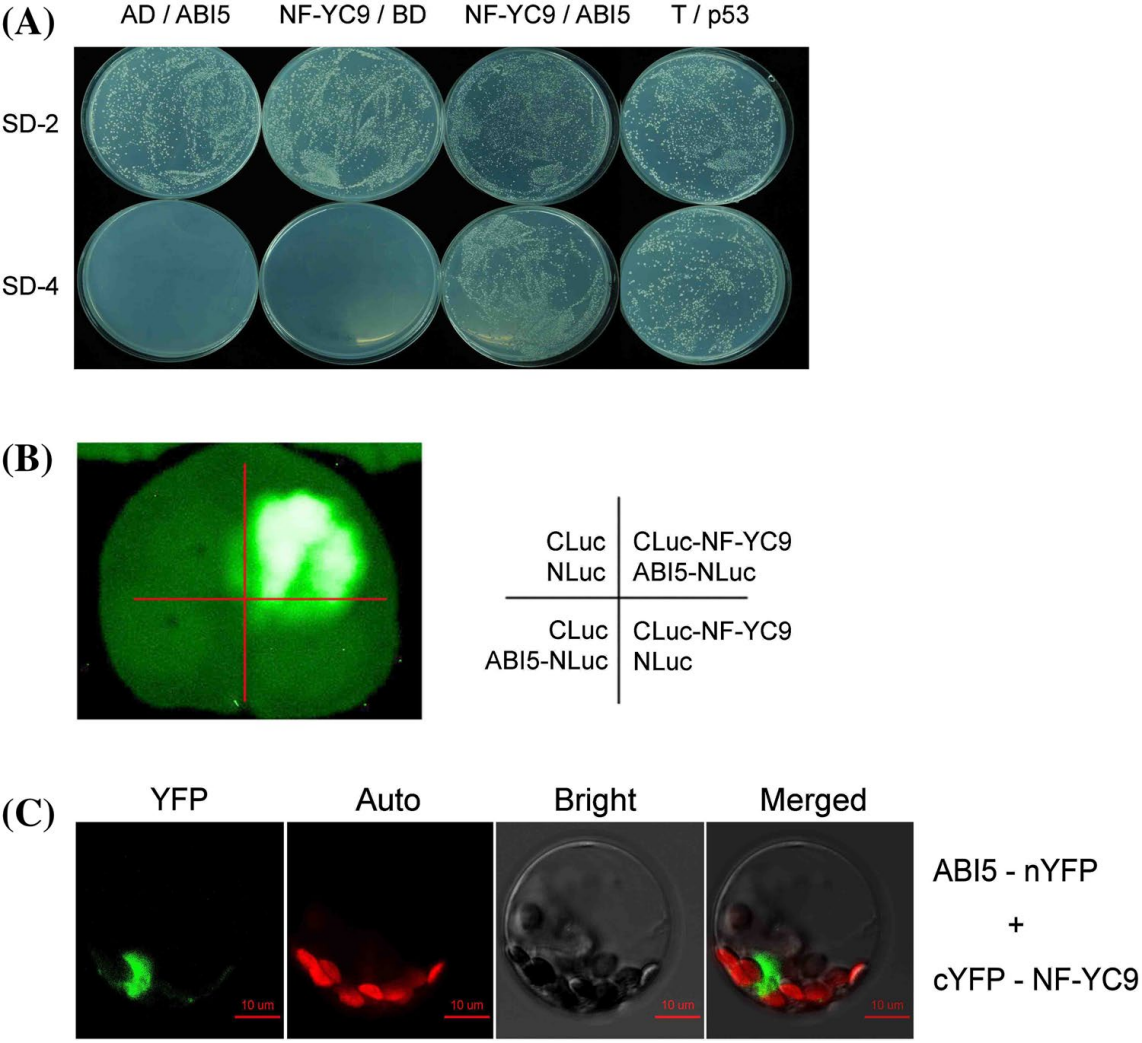
NF-YC9 interacts with ABI5. **a** Assays of yeast two-hybrid growth in SD4-drop-out medium (lacking Leu, Trp, His, and Ade) to test the interaction between NF-YC9 and ABI5 protein. The full-length coding sequence of *NF-YC9* was cloned to pGADT7 vector fused with the AD, and the full-length coding sequence of *ABI5* was cloned to pGBKT7 vector fused with the BD. The yeast cells co-transformed with the construct pairs AD-NF-YC9 plus BD-ABI5 or BD-53 plus AD-T (a positive control) were able to grow in the SD4-dropout medium (lacking Leu, Trp, His, and Ade), while the yeast cells co-transformed the construct pairs AD plus BD-ABI5 and BD plus AD-NF-YC9 (as negative controls) were not able to grow in the SD4-drop-out medium. The experiments were repeated three times with similar results. **b** Test of the interaction between NF-YC9 and ABI5 by LCI assay. The full-length coding sequence of *NF-YC9* and *ABI5* were constructed to the C-terminus of Luc (CLuc) and the N-terminus of Luc (NLuc) vector, respectively. Tobacco leaves were co-transformed with the construct pairs as described in the right panel. The left panel shows the luciferin fluorescence of the treated leaf. The experiments were repeated three times with similar results. **c** BiFC assay to test the interactions between NF-YC9 and ABI5 in the Arabidopsis protoplast system. The full-length coding sequence of *NF-YC9* and *ABI5* were constructed to the C-terminus of YFP (cYFP) and the N-terminus of YFP (nYFP) vector, respectively, and the construct pair was used to co-transform the protoplasts. The fluorescent signal was investigated by a confocal laser scanning microscope. YFP, ABI5-nYFP plus cYFP-NF-YC9 signal; Auto, chlorophyll auto-fluorescent signal; Bright, bright field; Merged, merged image of the YFP signal with chlorophyll auto-fluorescent signal in the bright field. The experiments were repeated three times with similar results

To test how NF-YC9 is involved in ABA signaling and based on bioinformatics analysis, we investigated whether NF-YC9 interacts functionally with an ABA-responsive transcription factor ABI5. For testing physical interaction between these two proteins in the yeast two-hybrid system, the full coding sequence of *NF-YC9* was cloned to pGADT7 vector fused with the AD, and the full coding sequence of *ABI5* was cloned to pGBKT7 vector fused with the BD. The yeast cells co-transformed with the construct pairs AD-NF-YC9 plus BD-ABI5 or BD-p53 plus AD-T (a positive control) were able to grow in the SD4-dropout medium (lacking Leu, Trp, His, and Ade), while the yeast cells co-transformed with the construct pairs AD plus BD-ABI5 and BD plus AD-NF-YC9 (as negative controls), were not able to grow in the SD4-dropout medium, indicating that NF-YC9 interacts with ABI5 and that the interaction detected in this yeast system is specific and reliable (Fig. 6a).

Next, we used the tobacco LCI system to confirm the interaction between NF-YC9 and ABI5. NF-YC9 and ABI5 were constructed to the C-terminus of Luc (CLuc) and the N-terminus of Luc (NLuc), respectively. NF-YC9-CLuc and ABI5-NLuc co-expressed tobacco leaves displayed significant Luc activity (strong fluorescence), but no Luc activity was detected in the leaves co-infiltrated with three negative control pairs (NLuc plus CLuc, NF-YC9-CLuc plus NLuc or CLuc plus ABI5-NLuc) (Fig. 6b).

We further tested this interaction in the Arabidopsis protoplast system with BiFC. NF-YC9 and ABI5 were constructed to the C-terminus of yellow fluorescent protein (cYFP) and the N-terminus of YFP (nYFP), respectively. The signal of yellow fluorescent (YFP) were detected by co-expression of cYFP-NF-YC9 with ABI5-nYFP (Fig. 6c). These data from both LCI and BiFC systems consistently verify the interaction between NF-YC9 and ABI5.

### NF-YC9 both facilitates ABI5 activity and enhances expression of *ABI5* gene in response to ABA

**Fig. 7:**
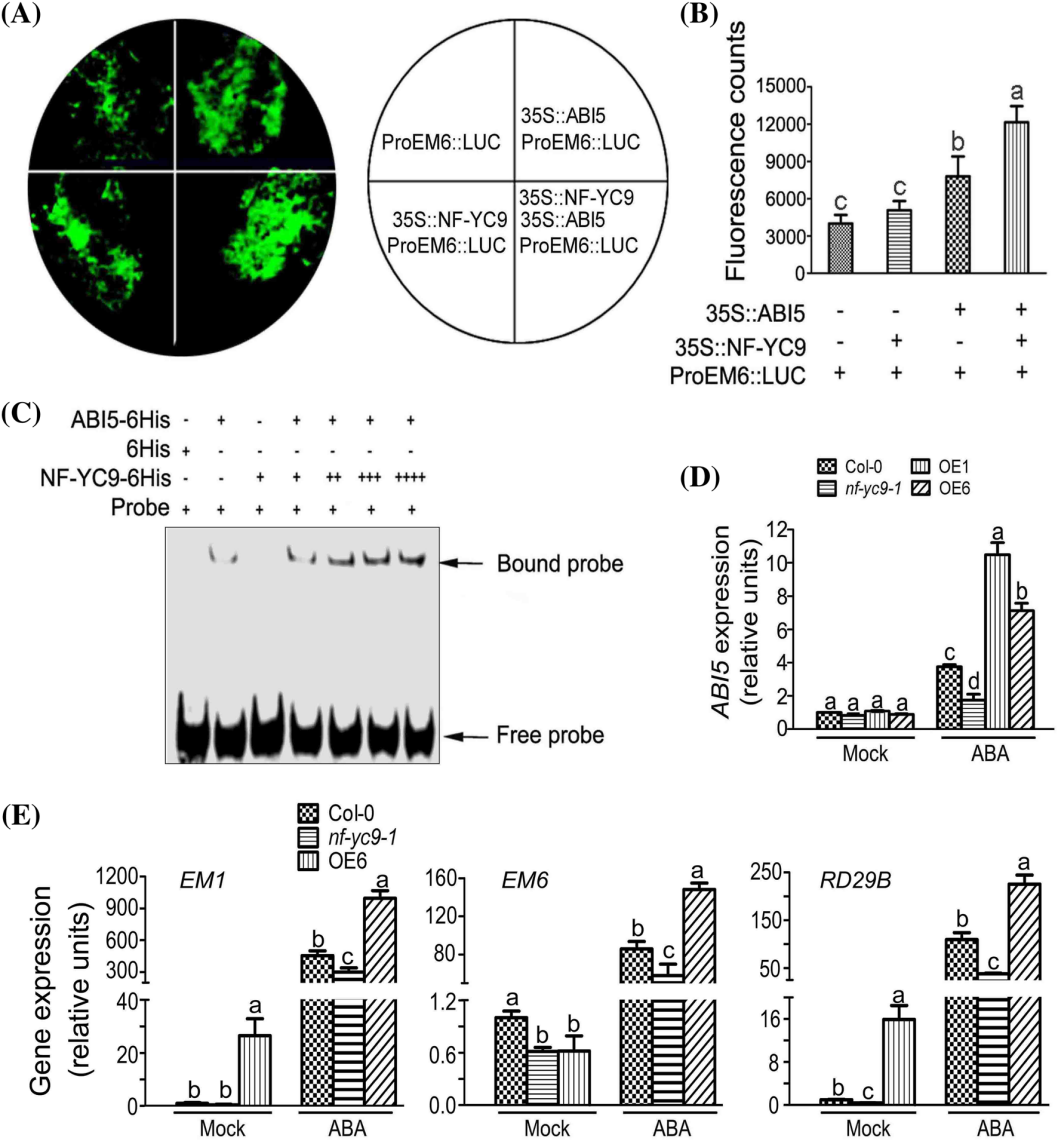
NF-YC9 enhances both activities of ABI5 transcription factor and expression of the *ABI5* gene. **a** Transient expression assays in *N. benthamiana* leaves, showing that NF-YC9 facilitates ABI5 to activate the target gene *EM6* transcription. Representative images of the *N. Benthamiana* leaves 60 h after infiltration are shown. Tobacco leaves were transformed with the construct combinations as described in the right panel. *ProEM6* promoter of the *EM6* gene, 35S, 35S promoter; LUC. The experiments were repeated three times with similar results. **b** Quantitative analyses of the luminescence intensity as shown in (**a**). The panels show the corresponding quantitative data corresponding to fluorescent images. Each value is the mean ± SE with three independent determinations. The symbols “+” and “−” represent that the protein was and was not expressed, respectively, in the tobacco leaves. Each value is the mean ± SE of three independent biological experiments, and different letters indicate significant differences at P < 0.05 (Duncan’s multiple range test). **c** Electrophoretic mobility shift assay, showing that NF-YC9 promotes ABI5 to bind the target gene *EM6* promoter. The ABI5 and NF-YC9 proteins were tagged with the 6His tag peptide (6His). The symbols “+” and “−” represent that the protein was and was not added, respectively, to the EMSA incubation buffer. The NF-YC9-6His protein was added, at a gradient of concentrations, to the EMSA incubation buffer (1 μg for +; 2 μg for ++; 3 μg for +++; 4 μg for ++++) to test possible NF-YC9-protein dose-dependence of the ABI5-*EM6* promoter interaction. The experiments were repeated three times with the same results. **d** NF-YC9 stimulates expression of the *ABI5* gene: quantitative RT-PCR analysis of the expression levels of *ABI5* in the wild-type Col-0, *nf-yc9-1* knockdown mutant and the *NF-YC9* overexpression transgenic lines OE1 and OE6 plants in response to ABA treatment. Seedlings of these different genotypes were sprayed with 100 μM (±)ABA solution or mock solution [0 μM (±) ABA solution, as a control], and sampled for gene expression analysis 4 h after the ABA treatment. The gene expression levels are normalized relative to the value of wild-type Col-0 plants treated with 0 μM (±) ABA treatment, which is taken as 1. Each value is the mean ± SE of three independent biological experiments, and different letters indicate significant differences at P < 0.05 (Student’s *t* test) when comparing values within the same treatment. **e** NF-YC9 stimulates expression of the *EM1, EM6* and *RD29B* genes: quantitative RT-PCR analysis of the expression levels of *EM1, EM6* and *RD29B* in the wild-type Col-0, *nf-yc9-1* knockdown mutant and the *NF-YC9* overexpression transgenic line OE6 plants in response to ABA treatment. Seedlings of these different genotypes were sprayed with 100 μM (±)ABA solution or mock solution [0 μM (±) ABA solution, as a control], and sampled for gene expression analysis 4 h after the ABA treatment. The gene expression levels are normalized relative to the value of wild-type Col-0 plants treated with 0 μM (±) ABA treatment, which is taken as 1. Each value is the mean ± SE of three independent biological experiments, and different letters indicate significant differences at P < 0.05 (Duncan’s multiple range test) when comparing values within the same treatment

To test functional significance of the NF-YC9-ABI5 interaction, we used a well-established transient expression assay in *N. Benthamiana* (Chen et al. 2011; Zhai et al. 2013), in which a direct downstream target gene of ABI5, *EM6* (Carles et al. 2002), was used as a candidate target. We constructed a vector of LUC fused with the *EM6* promoter from the start codon ATG to the upstream 1973 bp (ProEM6::LUC reporter). The full coding sequence of the *NF-YC9* and *ABI5* genes were fused to the pMDC85 and pCAMBIA1300-221 expression vectors as the effector constructs (35S::NF-YC9, 35S::ABI5), respectively. Co-expression of ProEM6::LUC with the 35S::ABI5 showed a significant increase of the luminescence intensity, indicating that 35S::ABI5 promoted the expression of ProEM6::LUC in this system as expected (Fig. 7a, b). Co-expression combination of 35S::NF-YC9, 35S::ABI5 and ProEM6::LUC enhanced significantly the luminescence intensity of the reporter compared with the co-expression of ProEM6::LUC with the 35S::ABI5 alone (Fig. 7a, b). This observation suggests that NF-YC9 facilitates the function of ABI5 that activates transcription of the *EM6* gene.

We further test this cooperative effect of NF-YC9 and ABI5 on the promoter activity of the target gene *EM6* in the EMSA system using purified ABI5-6His and NF-YC9-6His recombinant proteins (with 6His tag protein as a negative control). Previous work (Carles et al. 2002) showed that ABI5 binds the *EM6* promoter sequence called the pEm6.2 probe (AATAAAGTCAGACACGTGGCATGTCACCAA) with the highest affinity. So we chose and synthesized the ATG upstream (−220 to −277 bp) fragment of *EM6* promoter region, which includes the pEm6.2 probe sequence and used as the probe in our experiment (by the gene synthesis company GBT, Beijing, China). We found that the sole ABI5-6His protein bound the probe, but the NF-YC9-6His protein did not bind the probe (Fig. 7c). The addition of a gradient of amounts of NF-YC9-6His fusion protein promoted the ability of ABI5-6His to bind the DNA probe in a dose-dependent manner (Fig. 7c), indicating that NF-YC9 improves the DNA binding ability of ABI5, consistent with the results from the above transient expression assay in *N. Benthamiana*.

Additionally and interestingly, we found that ABA-induced up-regulation of *ABI5* expression was significantly repressed in *nf-yc9-1* mutant, but enhanced in *NF-YC9*-overexpressing lines (OE1 and OE6) (Fig. 7d), suggesting that NF-YC9 both facilitates ABI5 activity and enhances expression of *ABI5* gene in response to ABA.

### *NF-YC9* expression level is positively correlated with expression levels of ABI5 target genes *EM1*and *EM6*

Given that NF-YC9 is ubiquitously expressed in all the organs/tissues with the highest level in dry seeds (Fig. 5b; see Supplemental Fig. S1 in Supplemental Data), it is likely that down-expression of *NF-YC9* and over-expression of *NF-YC9* would affect transcript levels of genes downstream of ABI5. Early studies indicated that many late embryogenesis abundant (LEA) genes such as *Em1* and *Em6* were *ABI5*-target genes, as their transcripts were significantly reduced in *abi5* mutants (Finkelstein and Lynch 2000; Carles et al. 2002). As expected, transcripts of *EM1* and *EM6* were significantly reduced in *nf-yc9-1* mutant, but enhanced in *NF-YC9*-overexpressing line OE6 (Fig. 7e). Besides, we found that ABA-induced up-regulation of *RD29B* expression (Shinozaki and Yamaguchi-Shinozaki 2007) was significantly repressed in *nf-yc9-1* mutant, but enhanced in *NF-YC9*-overexpressing line OE6 (Fig. 7e), indicating that other ABA-inducible genes are also likely regulated by NF-YC9. Consistent with the above mentioned data, these observations support the idea that NF-YC9 positively regulates ABI5 transcription activity.

## Discussion

### NF-YC9 may be involved in ABA signaling potentially as a positive regulator

As described above, nuclear factor Y (NF-Y) family proteins NF-YA, NF-YB and NF-YC subunits may function to directly regulate gene expression together as different hetero-trimers or alone as a single NF-Y subunit without formation of a hetero-trimer (Gusmaroli et al. 2001; Stephenson et al. 2007; Siefers et al. 2009; Petroni et al. 2012; Laloum et al. 2013; Shi et al. 2014). They may also function to interact with and help other transcription factors by working together or alone (Wright et al. 1995; Benatti et al. 2008; Yamamoto et al. 2009; Liu and Howell 2010; Kumimoto et al. 2013; Yotsui et al. 2013; Yeap et al. 2017). Some NF-Y members are also involved in chromatin remodeling to modulate gene expression (Cao et al. 2014; Hou et al. 2014; Tang et al. 2017). This complicated mode of action likely allows the functional diversity and complexity of the NF-Y family proteins. Indeed, many members of NF-YA, NF-YB and NF-YC subfamilies have been shown to be involved, positively or negatively, in ABA and stress signaling (Nelson et al. 2007; Warpeha et al. 2007; Li et al. 2008, 2013; Yamamoto et al. 2009; Liu and Howell 2010; Leyva-González et al. 2012; Kumimoto et al. 2013; Sato et al. 2014; Shi et al. 2014; Siriwardana et al. 2014). Among the NF-YC members, whether NF-YC9 is involved in ABA and stress signaling is unclear, because a previous report showed no related phenotypes with a single mutant of *NF-YC9* gene, *nf-yc9*, in seed germination (Kumimoto et al. 2013). However, an overexpression line of *NF-YC9* has been reported to be ABA hypersensitive in seed germination, as evidenced by lower testa and endosperm rupture rate (Liu et al. 2016). Especially, the seed germination of the *nf-yc3 nf-yc9* double mutant and *nf-yc3 nfyc4 nf-yc9* triple mutant were insensitive to ABA, whereas, surprisingly, the germination of the single mutant *nf-yc4* and double mutants *nf-yc3 nfyc4* and *nfyc4 nf-yc9* was hypersensitive to ABA (Kumimoto et al. 2013). Therefore it is confusing whether and how these NF-YC members regulate ABA signaling.

In the present experiment, we confirmed that down-regulation of *NF-YC9* has no effect on ABA response in seed germination (data not shown), which was consistent with the previous report (Kumimoto et al. 2013). Importantly, we observed that, while down-regulation of *NF-YC9* affects neither early seedling growth nor stomatal movement in response to ABA, over-expression of the *NF-YC9* gene confers ABA hypersensitivity in both early seedling growth and stomatal response (Figs. 1, 2). We also showed that over-expression of the *NF-YC9* gene confers salt and osmotic hypersensitivity in early seedling growth (Figs. 3, 4), which is likely to be directly associated with the ABA hypersensitivity. In fact, it is well known that ABA accumulates in salt stress as in other abiotic stresses, and increased levels of ABA result in inhibition of seed germination and are required for tolerance of seedling growth to salt (Zhu 2002, 2003; Shinozaki et al. 2003). Therefore, the salt and osmotic hypersensitivity resulting from *NF-YC9* over-expression in post-germination growth should be attributed to ABA hypersensitivity in the situation of the salt- and osmotic-induced high levels of ABA. This point of view may be supported by the observations that the *abi1 abi2* double knockout mutant, hypersensitive to ABA, is also hypersensitive to salt stress in seed germination and post-germination growth (Jiang et al. 2015). The same phenomenon was observed in other mutants (Achard et al. 2006). All our data suggest that NF-YC9 may be involved in ABA signaling potentially as a positive regulator, and likely functions redundantly together with other NF-YC members.

### How does NF-YC9 work in ABA signaling?

It has been shown that the plant NF-Y family proteins interact with other transcription factors, which regulate the downstream gene expression, especially those in the bZIP family (Wright et al. 1995; Benatti et al. 2008; Yamamoto et al. 2009; Liu and Howell 2010; Kumimoto et al. 2013). The *Arabidopsis* NF-YB9/LEC1 and NF-YB6/L1L were shown to interact with bZIP67 to regulate expression of the genes with ABA-responsive elements (ABRE) in their promoters to function in embryogenesis (Yamamoto et al. 2009). In the moss (*Physcomitrella patens*), NF-YC1 interacts with the ABA-responsive transcription factor ABI3 to form a complex to activate the *LEA1*, a target gene of ABI3 (Yotsui et al. 2013). In *Arabidopsis*, three NF-YC members NF-YC3, NF-YC4 and NF-YC9 interact physically with the bZIP transcription factors ABF1 to ABF4 and HY5 protein (Kumimoto et al. 2013), which suggests that the NF-YC members may function together with these bZIP transcription factors. However, it has been unknown whether these interaction complexes are associated with regulation of promoter activity or target gene expression. Most recently, *Arabidopsis* NF-YC3, NF-YC4 and NF-YC9 were shown to interact with the DELLA protein RGL2 (a key repressor of GA signaling), forming a NF-YC-RGL2 complex to module ABI5 and thus to regulate ABA signaling in seed germination (Liu et al. 2016). In these events of cell signaling, NF-YC-RGL2 complex functions together as a whole to target the promoter of *ABI5* gene, in which RGL2, not itself, but via NF-Y complex, recognizes ABI5 promoter region (Liu et al. 2016). In the present experiment, we showed that NF-YC9 physically interacts with the ABA-responsive bZIP transcription factor ABI5, and facilitates the function of ABI5 to bind and activate the expression of a target gene *EM6* (Figs. 6, 7). The expression analysis showed that NF-YC9 also positively regulates expression of another target gene *EM1* of ABI5 as well as an important ABA-responsive gene *RD29B* (Fig. 7). These data support the model that the NF-YC9, like NF-YB9 and NF-YB6 (Yamamoto et al. 2009) or the moss NF-YC1 (Yotsui et al. 2013), mediates ABA signaling via targeting to and aiding ABA-responsive transcription factors. Our data reveal that NF-YC9 interacts, not only with DELLA protein RGL2 to indirectly regulate ABI5 as previously described (Liu et al. 2016), but also directly with ABI5 itself to modulate function of ABI5, suggesting a different, additional model by which the NF-YC members function to regulate ABI5 in ABA signaling pathway. Noteworthily, we showed that down-regulation of NF-YC9 represses, but overexpression of NF-YC9 enhances, ABA-induced *ABI5* expression (Fig. 7d), supporting the idea that NF-YC9 both facilitates ABI5 activity and interacts with other components such as RGL2 (Liu et al. 2016) to directly target to and stimulate *ABI5* gene expression in response to ABA. The present findings help to understanding the functional mechanism of the NF-YC proteins in highly complex ABA signaling pathway.

## Electronic supplementary material

Below is the link to the electronic supplementary material.

Supplementary material 1 (PDF 175 KB)
