# Supplementary material for: Overexpression of the transcription factor NF-YC9 confers abscisic acid hypersensitivity in Arabidopsis
Source: Plant Mol Biol. 2017 Sep 18;95(4):425–39. doi: 10.1007/s11103-017-0661-1 (PMC5688200; doi:10.1007/s11103-017-0661-1)
Supplement: Supplementary file 1 — Supplementary material 1 (PDF 175 KB) [file 11103_2017_661_MOESM1_ESM.pdf]

Supplemental Data

Bi C et al. Overexpression of the transcription factor NF-YC9 confers abscisic acid hypersensitivity in *Arabidopsis*

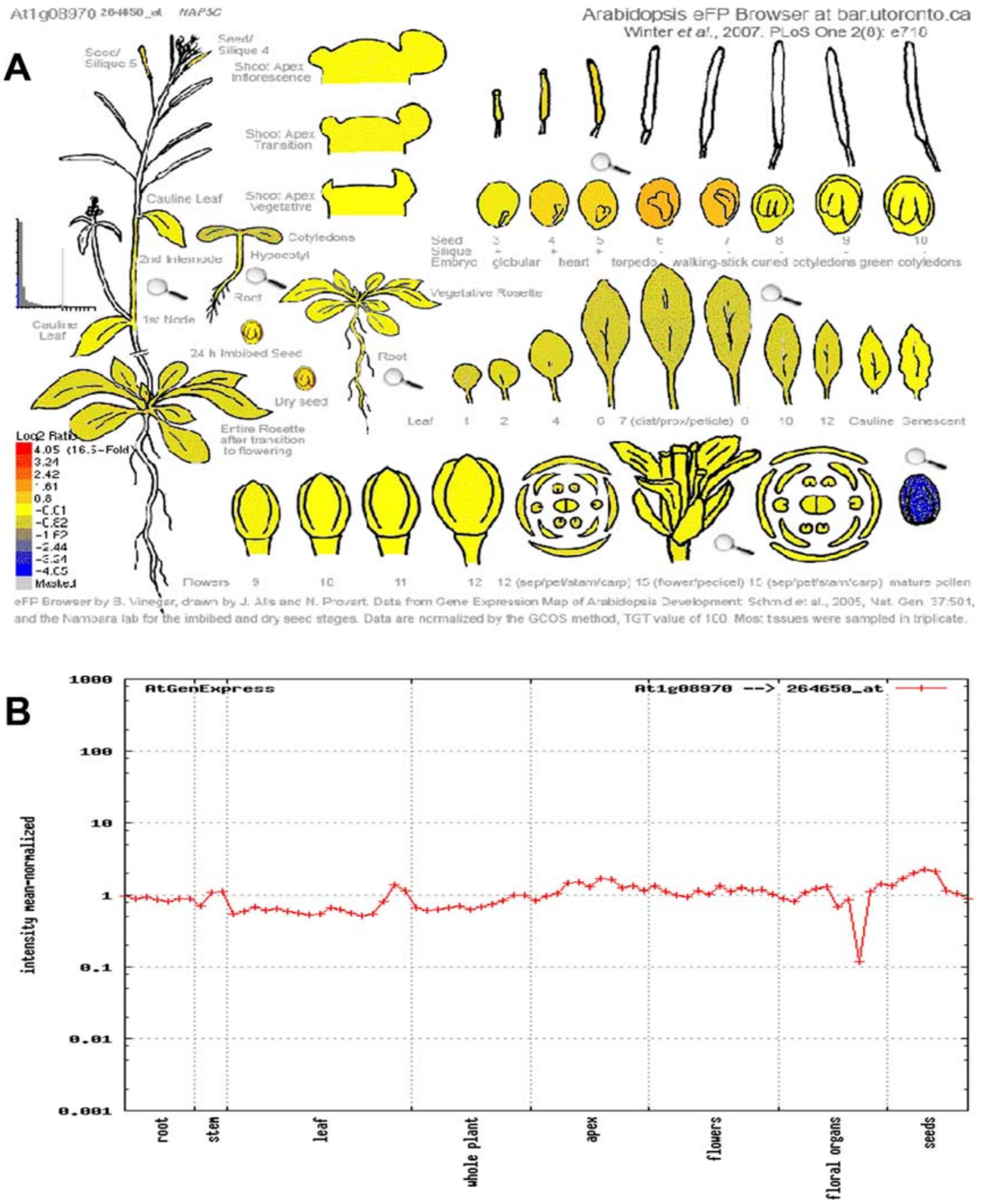

Supplemental Fig. S1 Expression of *NF-YC9* in various tissues/organs

(A) Expression of *NF-YC9* in various tissues/organs published at Arabidopsis eFP Browser (http://bar.utoronto.ca/efp/cgi-bin/efpWeb.cgi?dataSource=Developmental_Map). (B) Expression of *NF-YC9* in various tissues/organs published at AtGenExpress (http://jsp.weigelworld.org/expviz/expviz.jsp).

Supplemental Table S1. Primers for identification of T-DNA insertion. LP, left primer; RP, right primer

nf-yc9-1 (SALK_058903)	LP:	5’- CTATTGGAGCAGTTCCTGCAG-3’
	RP:	5’- AACCAGTCTCTTCCCCTTCAG-3’
nf-yc9-2(SALK_069632)	LP:	5’- AACTGCCGGTCATTGTTGTAG-3’
	RP:	5’- TTAAAAGCTGGGTGTCGTGTC-3’

Supplemental Table S2. Primers for assaying the expression levels of Col-0, *nf-yc9-1*, *nf-yc9-2* and NF-YC9 overexpression lines by RT-PCR. LP, left primer; RP, right primer

NF-YC9	LP-1:	5’- TTCCACCAGATCCATCAGCA-3’
	RP-1:	5’-TCCGCCTCTTATTCTCCTCA-3’
	LP-2:	5’-GATGAAGATGTCCGTATGAT -3’
	RP-2:	5’- AAATATCGGTTCTAGTCACA-3’

Supplemental Table S3. Primers for analysis of protein interaction by Yeast Two-Hybrid System

NF-YC9-AD	Forward primer:	5’- CGGAATTCATGGATCAACAAGACCATGG -3’
	Reverse primer:	5’- CGGGATCCCTAATTTTCCTGGTCAGGTT-3’
ABI5-BD	Forward primer:	5’- GGAATTCATGGTAACTAGAGAAACGAA -3’
	Reverse primer:	5’- CGGGATCCTTAGAGTGGACAACTCGGGT-3’

Supplemental Table S4. Primers for electrophoretic mobility shift assay (EMSA)

NF-YC9-6His	Forward primer:	5’- ACGCGTCGACATGGATCAACAAGACCATGG -3’
	Reverse primer:	5’-CGGAATTCGTGATGATGATGATGATGATTTTCCTGGTCA GGTTGGT-3’
ABI5-6His	Forward primer:	5’- ACGCGTCGACATGGTAACTAGAGAAACGAA -3’
	Reverse primer:	5’-CGGAATTCGTGATGATGATGATGATGGAGTGGACAACTC GGGTTCC-3’
Probe-EM6-Biotin	Forward primer:	ACCACGTTCAAAGTATACGTCAGCTAATAAAGTCAGACAC GTGGCATGTCACCAAGAA
	Forward primer:	TTCTTGGTGACATGCCACGTGTCTGACTTTATTAGCTGACGT ATACTTTGAACGTGGT

Supplemental Table S5. Primers for luciferase complementation imaging assay (LCI)

NF-YC9-CLuc	Forward primer:	5’- GGGGTACC ATGGATCAACAAGACCATGG -3’
	Reverse primer:	5’-ACGCGTCGACCTAATTTTCCTGGTCAGGTT -3’
ABI5-NLuc	Forward primer:	5’- GGGGTACC ATGGTAACTAGAGAAACGAA -3’
	Reverse primer:	5’-ACGCGTCGAC GAGTGGACAACTCGGGTTCC-3’

Supplemental Table S6. Primers for assaying cis-activation of EM6 promoter activity by ABI5 in tobacco leaves

ProEM6::LUC	Forward primer:	5’-CTGCAGAACCAATGCATTGGAGTGATCTATATTATGCGCC-3’
	Reverse primer:	5’- ACGCGTCGACGGCTCTTTAGTTACTTACAC -3’
35::NF-YC9	Forward primer:	5’- GTTAATTAAATGGATCAACAAGACCATGG -3’
	Reverse primer:	5’- AGGCGCGCCAATTTTCCTGGTCAGGTTGGT -3
35S::ABI5	Forward primer:	5’- CCCAAGCTT ATGGTAACTAGAGAAACGAA -3’
	Reverse primer:	5’- GGGGTACCGAGTGGACAACTCGGGTTCC -3

Supplemental Table S7. Primers for BiFC assays

NF-YC9-cYFP	Forward primer:	5'- GCTCTAGAATGGATCAACAAGACCATGG -3'
	Reverse primer:	5'- ACGCGTCGAC ATTTTCCTGGTCAGGTTGGT -3'
ABI5-nYFP	Forward primer:	5'- TTGGCGCGCC ATGGTAACTAGAGAAACGAA -3'
	Reverse primer:	5'- ACGCGTCGAC GAGTGGACAACTCGGGTTCC -3'

Supplemental Table S8. Primers for subcellular localization of NF-YC9 protein

NF-YC9-GFP	Forward primer:	5'- CCGGAATTCATGGATCAACAAGACCATGG -3'
	Reverse primer:	5'- ACGCGTCGACGGATTTTCCTGGTCAGGTTGGT -3'
PYR1-RFP	Forward primer:	5'- CCTTAATTAAATGCCTTCGGAGTTAACACC -3'
	Reverse primer:	5' -AGGCGCGCCACGTCACCTGAGAACCACTTC -3'

Supplemental Table S9. Primers for assaying the *ABI5*, *EM1*, *EM6* and *RD29B* expression levels in wild-type Col-0, *nf-yc9-1* mutant and NF-YC9 overexpression lines by RT-PCR. LP, left primer; RP, right primer

ABI5	LP:	5'- CAATAAGAGAGGGATAGCGAACGAG -3'
	RP:	5'- CGTCCATTGCTGTCTCCTCCA -3'
EM1	LP:	5'- CAAAGCAACTGAGCAGAGAAGAGC -3'
	RP:	5'- CCTCCCTTGCTCCTTCCTTCA -3'
EM6	LP:	5'- CAGCAGATGGGACGCAAAGG -3'
	RP:	5'- TATTACATCCGTGTGGGGAAGTTTG -3'
RD29B	LP:	5'- CTTGGCACCACCGTTGGGACTA -3'
	RP:	5'- TCAGTTCCCAGAATCTTGAACT -3'
